# Automatic Screening of Pediatric Renal Ultrasound Abnormalities: Deep Learning and Transfer Learning Approach

**DOI:** 10.2196/40878

**Published:** 2022-11-02

**Authors:** Ming-Chin Tsai, Henry Horng-Shing Lu, Yueh-Chuan Chang, Yung-Chieh Huang, Lin-Shien Fu

**Affiliations:** 1 Department of Pediatrics Taichung Veterans General Hospital Taichung Taiwan; 2 Institute of Statistics National Yang Ming Chiao Tung University Hsing-chu Taiwan; 3 Institute of Electrical & Control Engineering, National Yang Ming Chiao Tung University Hsing-chu Taiwan; 4 Department of Pediatrics National Yang Ming Chiao Tung University Taipei Taiwan; 5 Department of Post-Baccalaureate Medicine, College of Medicine, National Chung Hsing University Taichung Taiwan

**Keywords:** transfer learning, convolutional neural networks, pediatric renal ultrasound image, screening, pediatric, medical image, clinical informatics, deep learning, ultrasound image, artificial intelligence, diagnostic system

## Abstract

**Background:**

In recent years, the progress and generalization surrounding portable ultrasonic probes has made ultrasound (US) a useful tool for physicians when making a diagnosis. With the advent of machine learning and deep learning, the development of a computer-aided diagnostic system for screening renal US abnormalities can assist general practitioners in the early detection of pediatric kidney diseases.

**Objective:**

In this paper, we sought to evaluate the diagnostic performance of deep learning techniques to classify kidney images as normal and abnormal.

**Methods:**

We chose 330 normal and 1269 abnormal pediatric renal US images for establishing a model for artificial intelligence. The abnormal images involved stones, cysts, hyperechogenicity, space-occupying lesions, and hydronephrosis. We performed preprocessing of the original images for subsequent deep learning. We redefined the final connecting layers for classification of the extracted features as abnormal or normal from the ResNet-50 pretrained model. The performances of the model were tested by a validation data set using area under the receiver operating characteristic curve, accuracy, specificity, and sensitivity.

**Results:**

The deep learning model, 94 MB parameters in size, based on ResNet-50, was built for classifying normal and abnormal images. The accuracy, (%)/area under curve, of the validated images of stone, cyst, hyperechogenicity, space-occupying lesions, and hydronephrosis were 93.2/0.973, 91.6/0.940, 89.9/0.940, 91.3/0.934, and 94.1/0.996, respectively. The accuracy of normal image classification in the validation data set was 90.1%. Overall accuracy of (%)/area under curve was 92.9/0.959..

**Conclusions:**

We established a useful, computer-aided model for automatic classification of pediatric renal US images in terms of normal and abnormal categories.

## Introduction

Renal abnormalities are important findings in pediatric medicine. It is well accepted that “silent” renal abnormalities can be effectively detected through ultrasound (US) screening, which makes both early diagnoses and intervention possible [1,2]. US is a safe, relatively cheap, and convenient medical modality. Portable ultrasonic probes and internet connections have largely developed in recent years, even extending the coverage of pediatric renal US screening throughout the world. However, current methods remain limited due to the lack of automated processes that accurately classify diseased and normal kidneys [3].

Common renal abnormalities identified in US images in a series of more than 1 million school children included hydronephrosis (39.6%), unilateral small kidney (19.8%), unilateral agenesis (15.9%), cystic disease (13.9%), abnormal shapes—ectopic, horseshoe, and duplication of kidney (8%)—as well as others, that is, stones, tumors, and parenchymal diseases (1.5%) [1].

Thus far, publications regarding computer-aided US image interpretation have been much fewer than those based on computerized tomography or magnetic resonance imaging [4,5]. The use of US presents unique challenges, such as different angles of image sampling, low image quality caused by noise and artifacts, high dependence on an abundance of operators, and high inter- and intra-observer variability across different institutes and manufacturers’ US systems [6]. From the review about medical US published in 2021 [7], there were only 3 studies involving deep learning for renal US image classification [5,8,9].

This study was performed to select normal pediatric renal US images, as well as different types of renal abnormalities previously mentioned, for purposes of machine learning. Through the pretreatment of original images, adequate grouping of images, and deep neural network training, we hope that renal images can be correctly classified as either normal or abnormal. The aim of this study is to establish an artificial intelligence (AI) model for screening renal abnormalities to enhance the well-being of children even in areas where there is no pediatric nephrologist.

## Methods

### Ethics Approval

This study was approved by the institutional review board of Taichung Veterans General Hospital (No. CE20204A).

### Materials

The images used were all created from the original images in the pediatric US examination room at Taichung Veterans General Hospital from January 2000 to December 2020. Here were 4 different US machines manufactured by both Philips and Acuson, which were used in this study. All images were obtained by a US technician having more than 20 years of experience, using a 4 MHz sector transducer. We chose only images taken of a longitudinal view from the right and left kidney.

We established 2 data sets. One data set was for training, and the other was for validation. The images in these 2 data sets were totally different.

### Image Preprocessing and Data Cleaning

**Figure 1:**
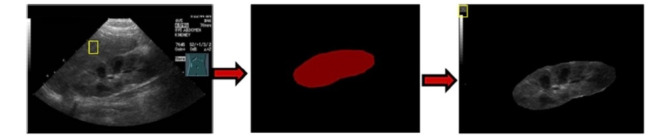
Preprocessing images for machine learning.

All images were detached from their original general data, including name, date of birth, date of examination, and chart number. The size of all the images was 600x480 pixels. We processed the images using software to obtain adequate illustrations for machine learning. As shown in Figure 1, after preprocessing, the images contain a kidney, a square of liver obtained from the examination simultaneously, and a gray scale gradient seen in the left upper part of the image.

### Image Grouping

Normal images were those having a normal size and shape, as well as a clear renal cortex or medulla without hydronephrosis, hyperechogenicity, cysts, stones, or any space-occupying lesion. We prepared 330 images for this group. There were a total of 1269 abnormal renal images. The abnormalities included hydronephrosis, hyperechogenicity, cysts, stones, and space-occupying lesions. The number of images and examinations are summarized in Table 1. The hyperechogenicity of the renal US images included increased renal cortex echogenicity as compared to the liver, a poor differentiation of the renal cortex or medulla, and an inversed echogenicity of the renal cortex or medulla. These findings were judged by 2 pediatric nephrologists.

**Table 1:** Distribution of images and examinations in the training and testing augmented database.

Diagnosis		Training (cases/images)	Testing (cases/images)	Totals (cases/images)
Normal		132/264	32/66	164/330
**Abnormal**				
	Stone	146/342	37/85	183/427
	Cyst	100/215	25/53	125/268
	Hyperechogenicity	60/132	15/33	75/165
	Space-occupying lesions	108/181	26/45	134/226
	Hydronephrosis	68/146	16/37	84/183
Total		614/1280	151/319	765/1599

### Machine Learning

We performed feature extraction with the pretrained model of ResNet-50 [8-10] in PyTorch from the data set ImageNet [11]. We used the pretrained weight of ResNet, so there was no backpropagation during feature extraction for training US images. The input data used were renal US images of 800x600 pixels in size. We normalized the dimension to 224x224 pixels prior to feeding the images into the network.

For the classification purpose, we redefined the final fully connected layers, which output image classification as abnormal or normal. After the training images went through Resnet50, there were 2048 outputs. There were 4 components in the final fully connected layer. The first was a linear layer with the 2048 feature extractions and 512 outputs. The second was rectified linear unit, which was a piecewise linear function that only outputted the positive result. Subsequently, we added the dropout layer to prevent overfitting. The 4th component was another linear layer, performing with 512 inputs and 2 outputs, which stand for the 2 categories, that is, abnormal and normal class with their probabilities.

**Figure 2:**
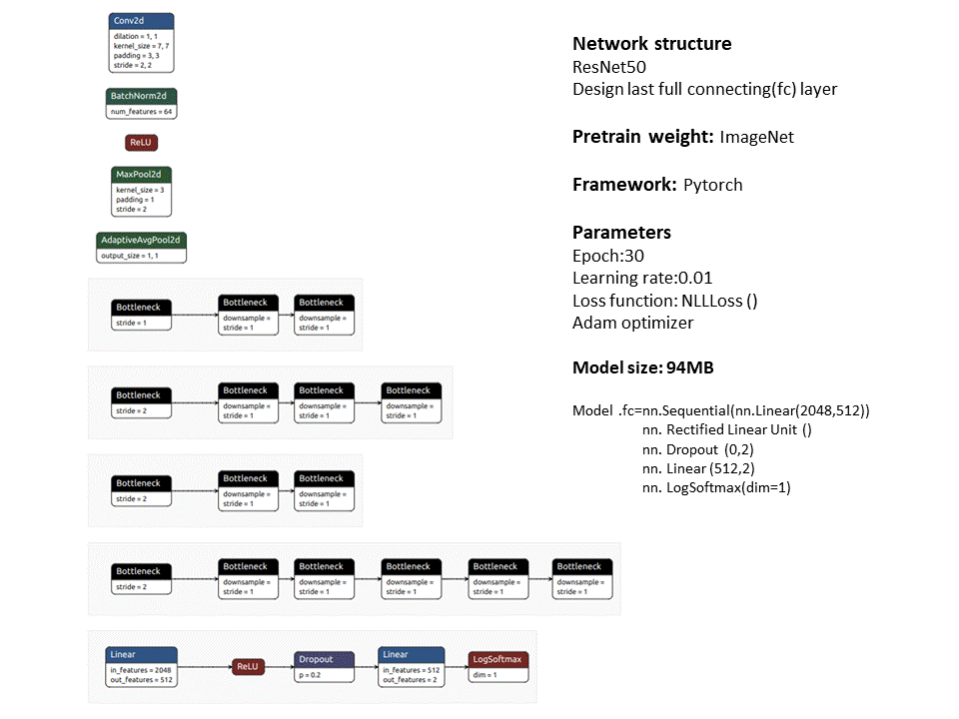
Brief summary of machine learning.

We optimized the model with the Adam optimizer at a learning rate of 0.01 [12]. There were a total of 30 epochs used for convolutional neural network training. We created a 94 MB size model to classify normal versus abnormal renal US images. Figure 2 is a summary of our deep learning structure.

### Experimental Setup

We implemented the training-testing approach. The data set was randomly divided into 1272/1599 (79.55%) images for training and 327/1599 (20.45% )images for testing to establish the model. We performed a 10-time randomization of the data set to repeat the machine learning described in the previous paragraph. For validation of the 94 MB model, there was another validation data set with 327 pediatric renal US images, including 66 (20.2%) normal, 37 (11.3%) hydronephrosis, 53 (16.2%) cyst, 95 (29.1%) stone, 53 (16.2%) hyperechogenicity, and 26 (7.9%) space-occupying US images. All these images were totally different from the data set for establishing the model.

### Evaluation of Performance

We evaluated the performance from a single image result. The diagnostic performance was measured by accuracy, specificity, sensitivity, positive predictive value, and negative predictive value. To calculate the above metrics, we defined an abnormal result as positive and a normal result as negative.

## Results

After 30 epochs for these 1599 pediatric renal US images, we obtained satisfactory results. The performance metrics in the test part of the data set are shown in Table 2. The accuracy in different abnormalities ranged from 95% to 100%.

**Table 2:** Evaluation metrics for screening different abnormalities from test renal ultrasound images in the data set.

Diagnosis (number)	Accuracy (%)	Sensitivity (%)	Specificity (%)	AUC-ROC^a^	PPV^b^ (%)	NPV^c^ (%)
Stone	100	100	100	0.974	100	100
Cyst	95.2	88.5	100	0.945	100	91.7
Hyperechogenicity	98.3	96.2	100	0.938	100	97.1
Space-occupying lesions	98.7	95.6	100	0.935	100	97.1
Hydronephrosis	100	100	100	0.998	100	100
Overall	98.4	96.39	100	0.961	100	97.2

^a^AUC-ROC: area under the receiver operating characteristic curve.

^b^PPV: positive predictive value.

^c^NPV: negative predictive value.

The accuracies of each abnormality ranged from 95.2% to 100%, with an overall accuracy as 98.4%. The area under curves (AUCs) were from 0.935 to 0.998. The AUC for overall performance was 0.961. There was no difference between these 10 random tests (*P*>.05). We repeated the 10 experiments using different randomizations involving 80%/20% training/test images to check the consistency of the machine learning performance. The accuracies ranged from 95.2% to 98.4%. There was no difference between these 10 tests (*P*>.05). We performed a 5-fold cross test, and the results are shown in Table 3.

**Figure 3:**
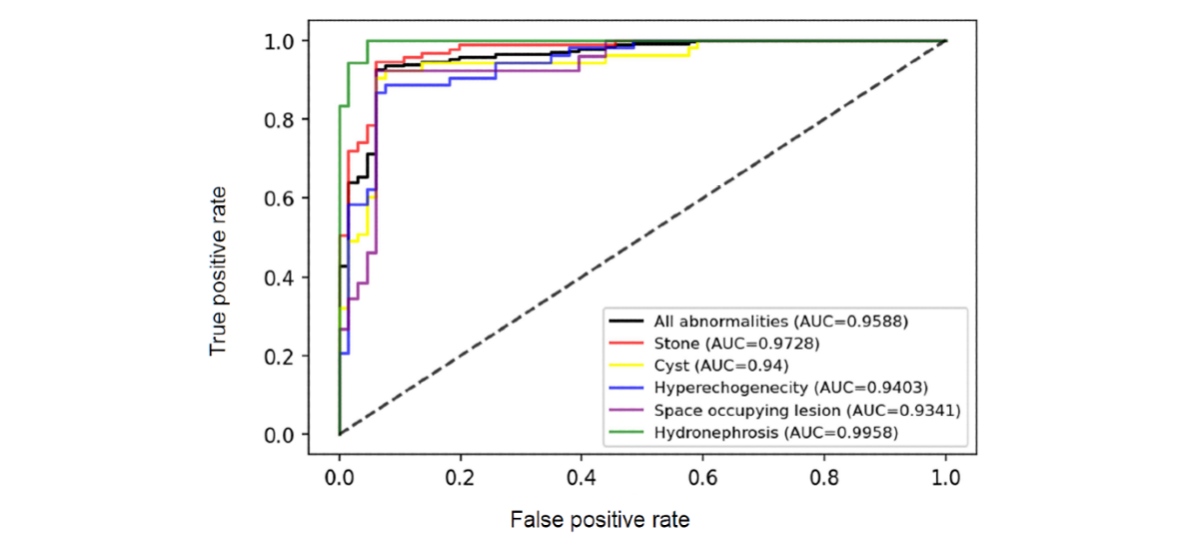
Area under the receiver operating characteristic curves of different image abnormalities and the overall performance. AUC: area under curve.

We validated the 94 MB model through machine learning with another 327 pediatric renal US images. The classifications included 66 (20.2%) normal, 37 (11.3%) hydronephrosis, 53 (16.2%) cyst, 95 (29.1%) stone, 53 (16.2%) hyperechogenicity, and 26 (7.9%) space-occupying US images. The performances based on each single image are summarized in Table 4. Accuracy in the different abnormalities ranged from 89.9% to 94.1%, with an average of 92.3%. AUC was from 0.934 to 0.996 (Figure 3). The overall performance in AUC was 0.959. The macro *F*_1_ was 0.924.

**Table 3:** Results of the 5-fold cross test.

	Test 1	Test 2	Test 3	Test 4	Test 5	Overall
Normal accuracy (%)	80	87.9	87.9	87.9	87.9	86.32
Stone accuracy (%)/AUC^a^	91.2/0.925	92.9/0.897	89.4/0.923	89.4/0.925	94.3/0.927	91.60/0.927
Cyst accuracy (%)/AUC	75.4/0.858	90.6/0.896	84.9/0.927	90.6/0.898	82.1/0.891	85.3/0.903
hyperechogenicity accuracy (%)/AUC	84.8/0.848	81.8/0.855	81.8/0.862	81.8/0.862	81.8/0.891	84.2/0.859
Space-occupying lesion accuracy (%)/AUC	92.5/0.903	84.9/0.881	94.5/0.917	83.0/0.874	82.6/0.863	86.8/0.896
Hydronephrosis accuracy (%)/AUC	100/0.965	91.9/0.888	89.2/0.940	94.6/0.932	91.4/0.871	94/0.928
Overall accuracy (%)/AUC	87.8/0.903	89/0.887	87.8/0.928	87.5/0.902	87.7/0.901	88.3/0.900

^a^AUC: area under curve.

**Table 4:** Evaluation metrics for screening different abnormalities from other renal ultrasound images for validation.

Diagnosis	US images, n (%)	Accuracy (%)	Sensitivity (%)	Specificity (%)	AUC-ROC^a^	PPV^b^ (%)	NPV^c^ (%)	*F*_1_-score
Normal	66 (20.2)	N/A^d^	N/A	90.9%	N/A	N/A	N/A	N/A
Stone	93 (28.4)	93.2	94.7	N/A	0.973	93.2	92.3	0.927
Cyst	53 (16.2)	91.6	92.5	N/A	0.940	91.6	93.8	0.918
Hyperechogenicity	53 (16.2)	89.9	88.7	N/A	0.940	89.9	90.9	0.897
Space-occupying lesions	26 (7.9)	91.3	92.3	N/A	0.934	91.3	96.81	0.923
Hydronephrosis	37 (11.3)	94.1	100	N/A	0.996	94.2	100	0.957
Overall	328 (100)	92.9	96.1	N/A	0.959	93.6	77.92	0.924^e^

^a^AUC-ROC: area under the receiver operating characteristic curve.

^b^PPV: positive predictive value.

^c^NPV: negative predictive value.

^d^N/A: not applicable.

^e^Macro *F*_1_.

## Discussion

The main finding of this study is a useful AI model for screening abnormal pediatric renal US images. The average accuracy can be 92.9%. The results can fulfill the main purpose of this study—to develop a useful computer-aided diagnosis model for screening various pediatric renal US abnormal patterns automatically. In this study, the machine learning methods were based upon convolutional neural network and fine-tuning, along with our unique methods for image preprocessing, as well as strategies for classification, which achieved a feasible model for clinical purposes. We constructed the stable classifier that combined both the transfer learning and training from scratch, balancing the training of a medical data set taken from an adequate sample size.

Clinical applications of AI in nephrology are versatile, but the use of renal US in this field is still in its infancy [13,14]. The reports derived from renal US images alone have been relatively limited up until now, with the major reports involving acute and chronic injuries [15-17]. Most renal image studies for AI used magnetic resonance imaging, computerized tomography, and patient histology for tumors, stones, nephropathy, transplantation, and other conditions [18-21]. The key challenges associated with deep learning involving US include reliability, generalizability, and bias [22]. The basic studies for enhancing AI performance in renal US have begun and remain undergoing [23-25].

There have been 4 reports from studies involving clinical AI applications in pediatric renal US abnormalities [3, 5,8,9]. Zheng et al [3] found that the deep transfer learning method offers satisfactory accuracy in identifying congenital anomalies in the kidney and urinary tract, even when the data set is as small as only having 50 children with congenital anomalies in the kidney and urinary tract and 50 children as the control. Yin et al [5] performed a similar study to detect posterior urethral valves. Sudarharson et al [8] used 3 variant data sets for identifying renal cysts, stones, and tumors, with an accuracy rate of 96.54% in images of quality and 95.58% in images of noise. Smail et al [9] attempted to use AI for grading hydronephrosis involving the 5-point scoring system from the Society of Fetal Urology (SFU). The best recorded performance was a 78% accuracy rate by dividing hydronephrosis into mild and severe. However, the accuracy rate was only 51% when using the 5-point system. In our study, we established a single 94 MB model to classify normal versus abnormal pediatric renal US images. The items seen in the abnormalities included renal cysts, stones, and tumors, as reported by Sudarharson et al [8]. In addition, the model was able to identify images of hydronephrosis and hyperechogenicity. Comparing the results from the study performed by Smail et al [9], our results showed a better classification accuracy for hydronephrosis. The 37 validated images were moderate or severe hydronephrosis, that is, the SFU class II, III, and IV. Our model can achieve 100% sensitivity, comparing the sensitivity of 46%-54%, as previously reported [26].

In terms of SFU class I, our model had an accuracy of 71.7% (119/166). Up until now, grading of hydronephrosis has been an ongoing challenge [27]. Extremely early intervention for treatment of mild hydronephrosis remains inadequate. If a child with mild hydronephrosis is also experiencing other renal abnormalities, such as stones, cysts, or hyperechogenicity, it is highly possible our model would be capable of providing any alarming information surrounding these conditions.

The unique pretreatment of images for machine learning performed in this study was performed to provide a comparison of liver echogenicity in the simultaneous study. This step is necessary for identifying hyperechogenicity. Other abnormalities, such as hydronephrosis, cysts, stones, and tumors, showed no difference in classification, regardless of whether we inputted the images with the addition of the square containing liver echogenicity and the gray scale gradient in the left part of the image shown in Figure 1. As demonstrated in Table 4, the accuracy and sensitivity for hyperechogenicity identification was lower than it was with other abnormalities. Increased echogenicity is an important finding in evaluating muscle, thyroid, vascular, and renal diseases [28]. The gray scale US presents a general sensitivity rate of 62% to 77%, a specificity of 58% to 73%, and a positive predictive value of 92% for detecting microscopically confirmed renal parenchymal diseases. The above results reveal that the echogenicity change was not sensitive enough for detecting renal disease. Abnormalities in renal echogenicity include increased echogenicity, poor differentiation of the cortex or medulla, and inversed echogenicity of the renal cortex and medulla [29]. In practice, it is quite often that we cannot obtain a square containing homogenous liver echogenicity for purposes of machine learning. When the classification is compared by a pediatric nephrologist, the results are acceptable. It is also difficult for the naked eye to discriminate between the not-so-significant gray scale differences. Currently, the so called “radiomics” information, which can aid US imaging in AI, is emerging [30], with a more precise assessment of US pixels possibly enhancing the utility of hyperechogenicity.

A limitation of this study is the single medical center image source. More images from different hospitals, areas, ethnicities, and US companies need to be used. We conducted a small-scale external validation using US images from different companies, including General Electric, Siemens, and Toshiba. After image pretreatment, the results could be 100% sensitivity, 80% specificity, and 90% accuracy. Another limitation is the moderate image number of images contributing to the data set. We did not divide images from right or left kidney for training, though the results can be acceptable. We will further validate our method based on larger data sets.

In conclusion, this study proposed the use of an automatic model for purposes of screening various abnormalities in pediatric renal US images. We will continue to enhance the model’s performance as we conduct additional evaluation studies surrounding its future clinical applications, including being an auxiliary software for screening children’s renal abnormalities in remote areas.

## Abbreviations

AI: artificial intelligence
AUC: area under curve
SFU: Society of Fetal Urology
US: ultrasound
